# Oscillations in the near-field feeding current of a calanoid copepod are useful for particle sensing

**DOI:** 10.1038/s41598-019-54264-1

**Published:** 2019-11-28

**Authors:** Carl Giuffre, Peter Hinow, Houshuo Jiang, J. Rudi Strickler

**Affiliations:** 1 Department of Mathematics and Computer Science, Adelphi University, Garden City, NY 11530 USA; 2 Department of Mathematical Sciences, University of Wisconsin - Milwaukee, Milwaukee, WI 53201 USA; 3 Department of Applied Ocean Physics and Engineering, Woods Hole Oceanographic Institution, Woods Hole, MA 02543 USA; 4 Department of Biological Sciences, University of Wisconsin - Milwaukee, Milwaukee, WI 53204 USA; 5 Marine Science Institute, University of Texas, Port Aransas, TX 78373 USA

**Keywords:** Behavioural ecology, Applied mathematics

## Abstract

Calanoid copepods are small crustaceans that constitute a major element of aquatic ecosystems. Key to their success is their feeding apparatus consisting of sensor-studded mouth appendages that are in constant motion. These appendages generate a feeding current to enhance the encounter probability with food items. Additionally, sensing enables the organism to determine the position and quality of food particles, and to alter the near-field flow to capture and manipulate the particles for ingestion or rejection. Here we observe a freely swimming copepod *Leptodiaptomus sicilis* in multiple perspectives together with suspended particles that allow us to analyse the flow field created by the animal. We observe a highly periodic motion of the mouth appendages that is mirrored in oscillations of nearby tracer particles. We propose that the phase shift between the fluid and the particle velocities is sufficient for mechanical detection of the particles entrained in the feeding current. Moreover, we propose that an immersed algal cell may benefit from the excitation by increased uptake of dissolved inorganic compounds.

## Introduction

Living organisms depend on extracting the “right” information from their environments. Carriers of informations are manifold: chemical substances announce the presence of food or conspecifics interested in mating, electromagnetic waves are the source of all vision, and mechanical signals in form of fluid disturbances allow to infer the presence of preys, predators, and again, conspecifics in both air and water. Planktonic copepods are one of the most abundant classes of animal life and occupy nearly every possible location in the worlds marine and freshwater bodies^1^. As they are located between autotrophic phytoplankton and higher heterotrophic organisms, they play a crucial role in the biogeochemical cycles on earth. Chemical and mechanical reception are their main way of obtaining information about their surroundings. They are alert swimmers and are known to create flow fields suited for different purposes. Fossilized copepods have been found in bitumen clast from the upper Carboniferous (303 Ma)^2^. Thus, we may be looking at the 500 millionth generation which gives a lot of room for evolutionary refinement of sensory appendages and the processing apparatus.

Life in water and with a body size of a few millimetres means “Life at Low Reynolds Number”^3^. To overcome the dominating viscosity due to the low Reynolds number, swimming and flying animals vibrate their appendages at high frequency, e.g. 30–70 Hz in planktonic copepods^4,5^. However, vibrating appendages create fluid mechanical signals that can be detected by predators at large distances^6–9^. Fish, the predators of planktonic copepods, sense the presence and location using their lateral line system^10^. Similar receptor systems have been described in various aquatic and terrestrial species, such as reptiles, amphibians, chaetognaths (arrow worms)^11^, insects^12^ and arachnids^13^. Legs of spiders are equipped with trichobothria that are capable of detecting oscillations of the air caused by potential prey several centimetres away. Cockroaches, crickets and mosquitoes have been found to respond to and to produce sound of selected frequencies, for example in order to ease mating^14,15^. The hairs and hair-like sensory organs in these arthropods are extremely sensitive, responding to displacements of a few nanometres^14,16^. Guided by the mechanosensory pathway in the central nervous system of the crayfish *Procambarus*^17^, we suggest that small velocity perturbations can also be captured by small aquatic arthropods.

Oscillatory movements of animal appendages (e.g., tails, fins, wings, cilia, and flagella) are a universal scheme for generating thrust for continuous propulsion by the animal in a fluid medium^18,19^. At low Reynolds number, in order to generate effective propulsion it is also required that the power stroke and the recovery stroke of the oscillatory movements should not be symmetric or reciprocal^3^. This is certainly true for planktonic animals, such as calanoid copepods^20^. Operating at low Reynolds number in the water, planktonic copepods oscillate their cephalic appendages achieve thrust for active swimming and to generate a feeding current, or more precisely, a scanning current^21,22^. Both active swimming and generation of a feeding current increase the encounter rate between the copepod and the suspended food items^23,24^. Copepod appendages are equipped with hair-like sensors (setae) with chemical and mechanical sensory functions^25–27^. The non-oscillatory far-field component of the feeding current enables the copepod to sense the presence, position and quality of food particles via chemoreception^8,28–30^. As the flow created by the copepod is chaotic in the vicinity of the animal, chemoreception is possible only for objects at greater distances. Near the animal, chemoreception has to be complemented by mechanoreception. Serendipitously, the oscillatory motion of the animal’s appendages needed for swimming, creates an oscillatory velocity field. Embedded particles that do not exactly match the specific weight of the surrounding water follow this oscillatory motion with a velocity shift. The velocity shift has a chance to be detected by the animal if it is above a certain threshold. Earlier observations and numerical simulations^30^ confirmed the ability of a copepod to detect streamline deformations by a particle in the direction of linear motion and to elicit a jump and capture reaction.

Here we present fluid-mechanical calculations to determine the phase shift between the velocities of an oscillating fluid and an immersed particle together with its maximal magnitude. Similar work has been recently done to determine the effect of the impulsive motion of a plate on a small sphere^31^. Accounting for the damping of the signal that emerges 200–300 *μ*m away from the sensory apparatus, we conclude that it is sufficient for detection of the particles entrained in the feeding current. Our theoretical considerations are complemented by observations of an adult freshwater calanoid copepod *Leptodiaptomus sicilis* swimming while sensing its environment for food. Earlier observations of small and translucent animals in water required that these be tethered to hairs to prevent the animals from escaping the field of vision of the camera. This may distort the behaviour of the animal as it may vigorously try to escape. In contrast, we observe a freely swimming copepod together with suspended particles at micrometer resolution. This technique was used recently in a study of the swimming behaviour of the copepod *Eurytemora affinis* under turbulent flow conditions^32^. The particles allow us to analyse the oscillatory flow field created by the animal. We observe a highly periodic motion of the mouth appendages that is mirrored in oscillations of the tracer particles in the adjacent regions. Our findings are compatible with a theory of echolocation in freshwater calanoid copepods. We also propose that algal particles in the feeding current stand to profit from exposure to turbulent shear.

## Results

### Observations of freely swimming copepods

**Figure 1:**
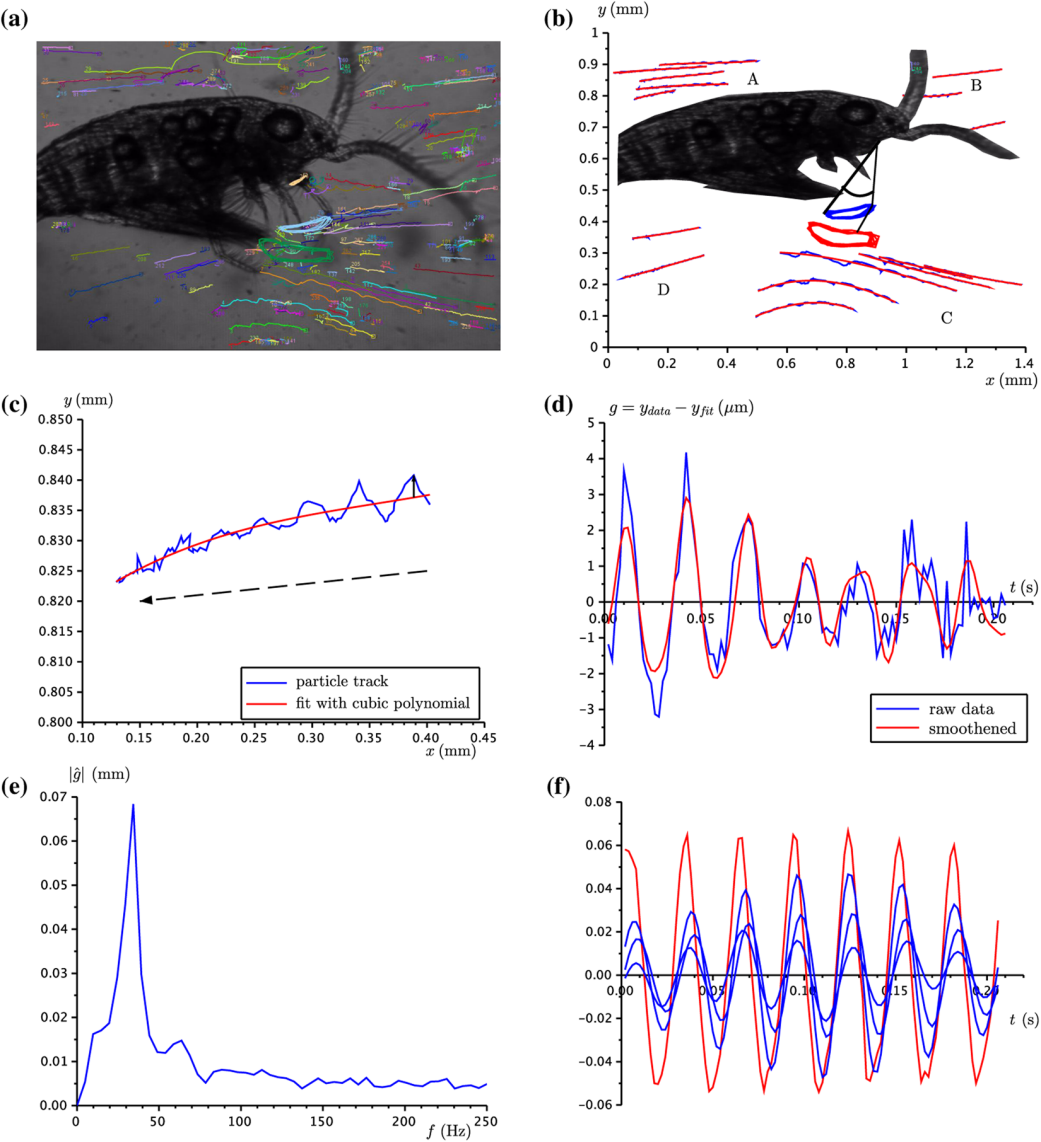
Overview of the animal and particle observations. **(a)** Lateral perspective of an untethered adult female copepod, its antennae, appendages and the immersed tracer particles (15 *μ*m diameter). **(b)** The oscillation of the appendage is defined by the trace of the first joint (closed blue curve) and the sweep angle is indicated. The particle tracks are grouped into four distinct regions A–D. **(c)** A sample trajectory of a particle from the dorsal posterior region A and the optimal fit with a cubic polynomial. The motion of the particle is in the direction of the arrow which is away from the copepod. The velocity of the underlying flow field is approximately 1.5 mm s^−1^. **(d)** The deviations from the centreline are used to determine the frequency of the oscillating flow field. Here the trajectory from panel **(c)** is used. **(e)** The averaged Fourier spectra of 15 particle trajectories from regions A, B and C. **(f)** Smoothened particle oscillations from region B (blue) and the oscillation angle of the maxilliped (red). For better visibility the particle oscillations have been amplified by a factor of 20.

**Figure 2:**
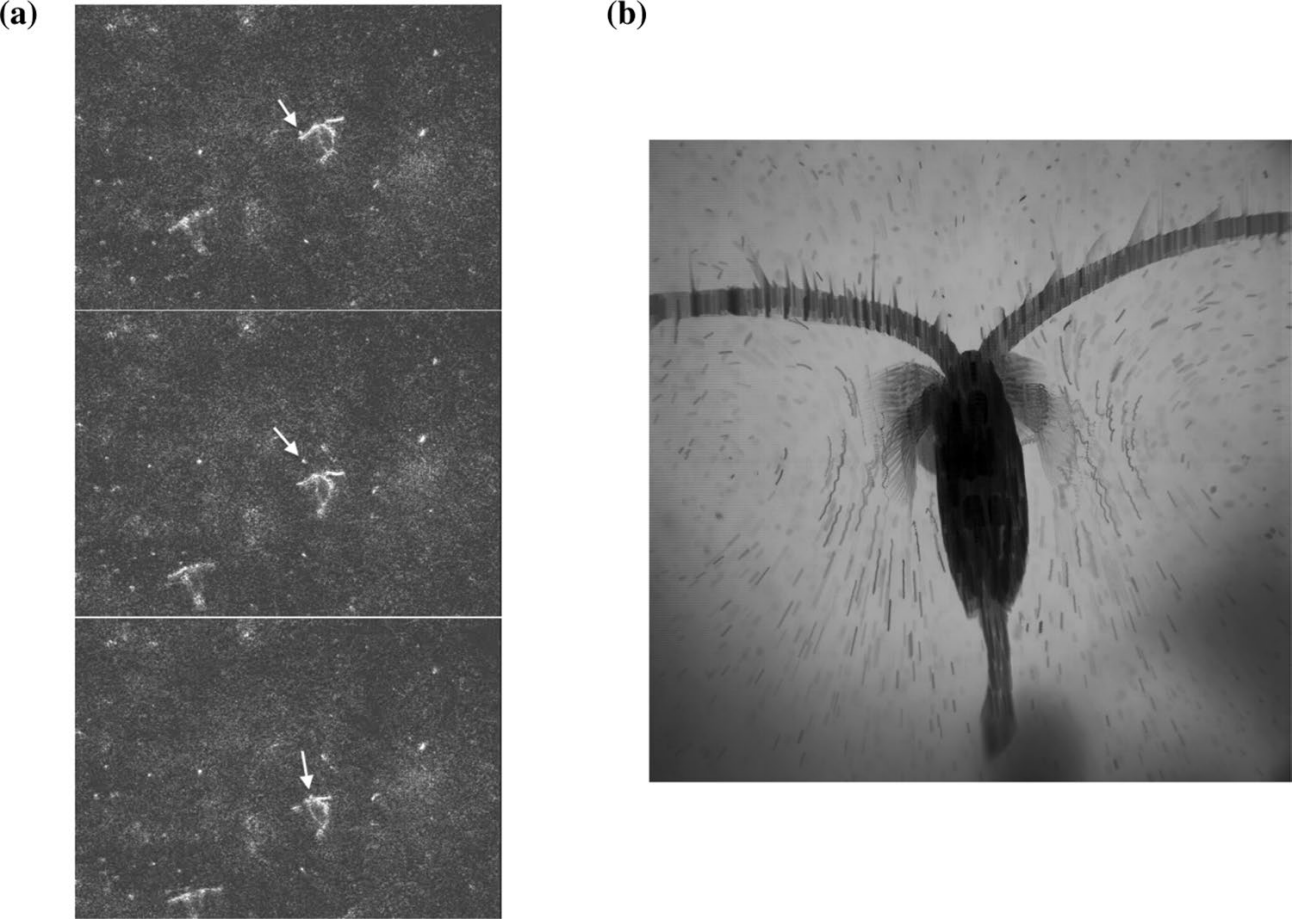
Further observations of untethered copepods and tracer particles. **(a)** Sequence of combined dorsal views (central area) and lateral views (lower left corner) of one animal and a particle of interest indicated by the arrow. Upon the encounter with the particle (top), the animal stops its beating motion to create some distance to the particle (centre) and then resumes the motion to consume the particle (bottom). **(b)** Dorsal view of an untethered *L. sicilis* and the tracer particles. The animal moves slightly during the 50 frames of the film. The particle oscillations are clearly visible and are superposed on the hourglass shaped flow lines.

A general perspective of an untethered copepod *Leptodiaptomus sicilis* is given in Fig. 1(a,b), together with designated regions in which the tracer particles are observed. In one film, the maxillipeds synchronously complete 7 cycles in 0.2 s, giving a frequency of 35 Hz (Supplementary Fig. S1(a)). The Fourier analysis reveals a dominant peak at 34 Hz and a much smaller peak at 68 Hz (Supplementary Fig. S1(b)). In the second part of the analysis, we fit cubic polynomials to the trajectories of the tracer particles and calculate the deviation of the actual trajectory from the fitted curve (Fig. 1(c,d)). Only those trajectories that do not intersect themselves are selected for further analysis. Trajectories in region D farther away from the copepod do not show oscillations. A total of 15 trajectories from all other regions show oscillations and are subjected to a discrete Fourier transform to reveal their frequency information. The averaged spectrum shows a peak at 34 Hz (Fig. 1(e)). After removal of noise, we plot the smoothened particle oscillations from different regions jointly with the appendage oscillations. Particle oscillations from the anterior region B are highly coherent (Fig. 1(f)) as are those from the dorsal posterior region A (Supplementary Fig. S2(a)). In contrast, trajectories from the ventral region C do not show a coherent pattern (Supplementary Fig. S2(b)). The coherence of particle oscillations in regions A and B and, by extension, the underlying flow field, suggests that most information is encoded in the oscillations in these regions. Note that particles in region B are naturally moving towards the animal and are likely the easiest to be picked up once they reach the vicinity of the mouthparts. Particles in the posterior dorsal region A are not lost to the copepod as it is able to change its motion pattern so that it recedes and the particles are eventually available for capture (Fig. 2(a)). In Fig. 2(b) we present a dorsal view of the same animal. We observe the particle oscillations superposed on the classical hourglass shaped flow field^33,34^ near the copepod.

### Embedded particles of differing density

If even small density differences between the particles and the fluid are present, the particles do not follow the flow entirely. In the following we compute the velocity of a tracer particle and its deviation from a surrounding oscillating flow field. The fluid velocity caused by the beating motion of the cephalic appendage with frequency *f* is given by

$${u}_{f}(t)=2\pi fA\,\cos \,(2\pi ft)$$

where the amplitude *A* (estimated from observations) is related to the animal prosome length *L* = 1 mm by *A* = 0.15⋅*L*. For an aspect ratio of the copepod of *s* = 0.37, according to^35^ the preferred food particle size is given by

$$d=\frac{L\cdot {s}^{\frac{2}{3}}}{18}=28\,\mu m.$$

The density ratio of the particle is

$$\gamma =\frac{{\rho }_{p}}{\rho }.$$

The kinematic viscosity of water at 20 °C is *ν* = 1.004·10^−6^ m^2^ s^−1^. Following [^36^, p. 307–308] we calculate

$$\begin{array}{rcl}\tau  & = & \frac{2\nu }{\pi f{d}^{2}},\,{H}_{2}=\frac{9}{2\gamma +1}\sqrt{\frac{\tau }{2}},\\ {H}_{1} & = & \frac{2(1-\gamma )/(2\gamma +1)}{{H}_{2}^{2}{(1+\sqrt{2\tau })}^{2}+{(1+{H}_{2})}^{2}},\\ {h}_{1} & = & {H}_{1}(1+{H}_{2}),\,{h}_{2}={H}_{1}{H}_{2}(1+2\tau ),\\ \beta  & = & \arctan \frac{{h}_{2}}{1+{h}_{1}},\,\eta =\sqrt{{(1+{h}_{1})}^{2}+{h}_{2}^{2}}\end{array}$$

where *τ* is the dimensionless period (or Stokes’ number) and *η* and *β* are the amplitude ratio and phase shift, respectively. Intuitively, for low frequency (high *τ*), the particle follows the fluid motion, as it does for very small deviations from the fluid density. The amplitude ratio and the phase shift determine the particle velocity

$${u}_{p}(t)=2\pi fA\eta \,\cos \,(2\pi ft+\beta ).$$

The maximum relative velocity between the particle and the local water motion is

1 $$m\,:=\,{\rm{\max }}({u}_{p}(t)-{u}_{f}(t))=2\pi fA\sqrt{{\eta }^{2}-2\eta \,\cos \,\beta +1}.$$

**Figure 3:**
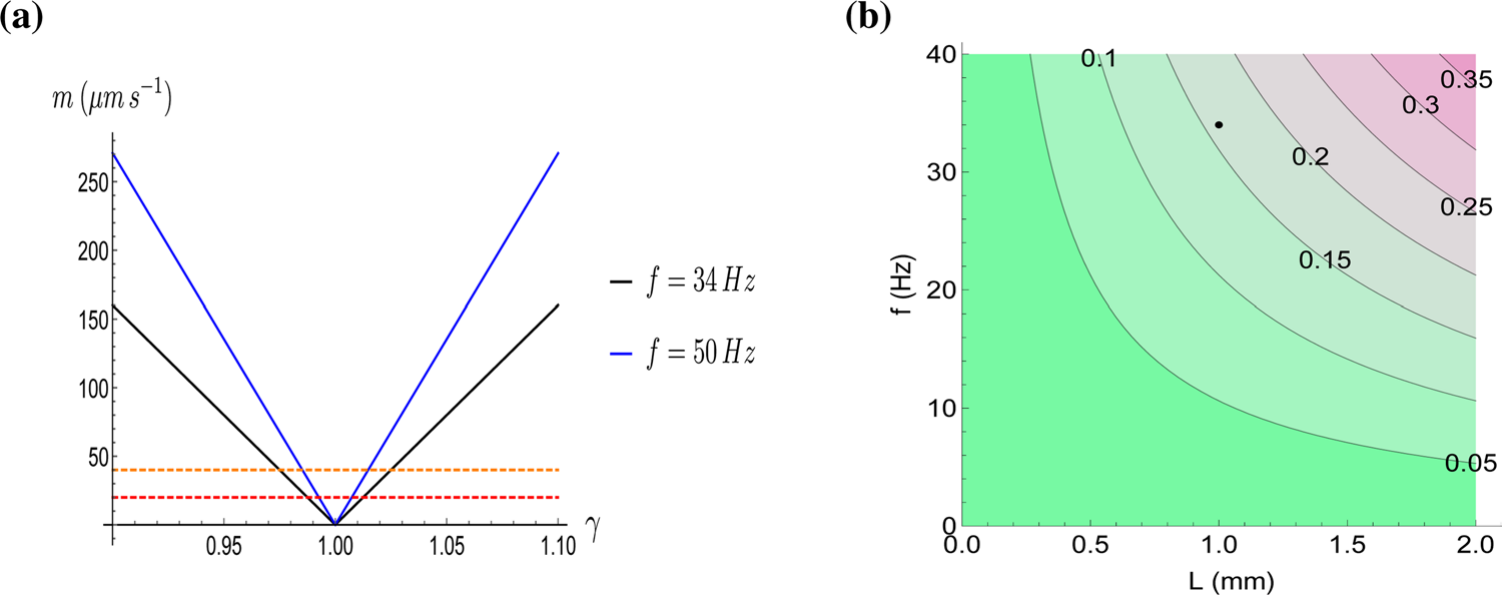
The maximal velocity difference of an immersed particle with a different density than water. **(a)** The maximal velocity difference *m* from Eq. (1) at the particle location as a function of the density ratio *γ* for two beating frequencies *f*. The dashed lines indicate possible detection thresholds of 20 and 40 *μ*m s^−1^ by the setae, respectively. However, the additional damping by the propagation through the water has o be factored in. **(b)** Contour plots of the maximal velocity difference *m* from Eq. (1) (in mm s^−1^) as function of prosome length *L* and beating frequency *f*. The density ratio is chosen to be *γ* = 1.1 while the remaining parameters are fixed to the same values as in the Results section. The black dot indicates the example *f* = 34 Hz.

We plot the maximum velocity difference as a function of the density ratio *γ* in Fig. 3(a) for selected beating frequencies.

A Stokes Boundary Layer is formed by a rigid plate oscillating with a frequency *ω* in a direction parallel to itself. The velocity disturbance decays exponentially with the distance from the plate. The thickness of the boundary layer is given by $\delta =\sqrt{2\nu /\omega }$ where again *ν* is the kinematic viscosity [^37^, p. 83–84]. Thus the penetration depth decreases with the frequency but increases with the kinematic viscosity of the fluid.

## Discussion

Located between autotrophs and heterotrophs in the aquatic food web, copepods play an important role and have been studied widely by ecologists. We have observed a freely swimming copepod *Leptodiaptomus sicilis* together with synchronously oscillating tracer particles in its vicinity. The 51 s long film can be considered a case of serendipity, as it is extremely difficult to find such an animal and to observe it for so long. A tethered copepod may sense that it is tethered and change its swimming behavior.

The maximal velocity difference from Eq. (1) is in many cases large enough for the copepod’s setae to detect. The densities of the cell sap and endoplasm of the green algae *Nitella*, *Nitellopsis* and *Chara* have been reported to be 1.01–1.015 g cm^−3^ ^38,39^ while the density of the cell wall is considerably larger, namely 1.35 g cm^−3^. Assuming a particle density of 1.05 g cm^−3^, the maximal velocity difference is approximately 160 *μ*m s^−1^ at the site of the particle. Ignoring the fact that we have a different geometry, the distance over which an oscillation at 34 Hz is damped by a factor of *e*^−1^ (≈37 %) is 240 *μ*m. This is within the distance of the particles in Fig. 1(a) to at least the nearest point on the surface of the copepod. An amplitude of 160 *μ*m s^−1^ would be damped to about 60 *μ*m s^−1^. This is above the detection threshold of 20* μ*m s^−1^ reported for the first antennae^16^.

In Fig. 3(b) we plot the contours of the expression (1) for varying prosome lengths *L* and beating frequencies *f*. Figures of this type go back to the early works of Schröder^5^, who found a functional relationship between body length and beating frequency in calanoid copepods. We propose that various copepod species of different lengths will select their beating frequencies to minimize the energetic cost but at the same time to make sure that the relative velocity differences are detectable. Many arthropod species are known to “listen” to external mechanical signals, and copepods have long been known to react to reflections of their own swimming wake to orient themselves^40^. The process of true echolocation differs in that the animal intentionally emits the outgoing signal, which is well known in insectivorous bats and toothed whales. The increased risk of the copepod itself being detected by its predators seems a price worth paying.

In this interaction, we have so far considered only one of the partners, the copepod. An algal particle is surrounded by a sphere that is enriched in organic molecules leaked by the cell. This phycosphere is the important interface in which exchanges between phytoplankton and bacteria and their surroundings take place^41–43^. As the flow around the cell changes from laminar to turbulent, the phycosphere gets deformed until it turns into a web of filaments, see e.g. Figure 3 in^42^. In such an event, the cell also leaves its “stale” diffusion limited surroundings and has access to fresh water with inorganic nutrients. Very recent research has demonstrated that cell chains of the diatom genera *Skeletonema* and *Chaetoceros* increased their carbon assimilation in turbulent shear conditions^44^. The bloom-forming and toxic cyanobacterium *Dolichospermum* is also able to increase its nutrient uptake in more livid hydrodynamical conditions^45^.

**Figure 4:**
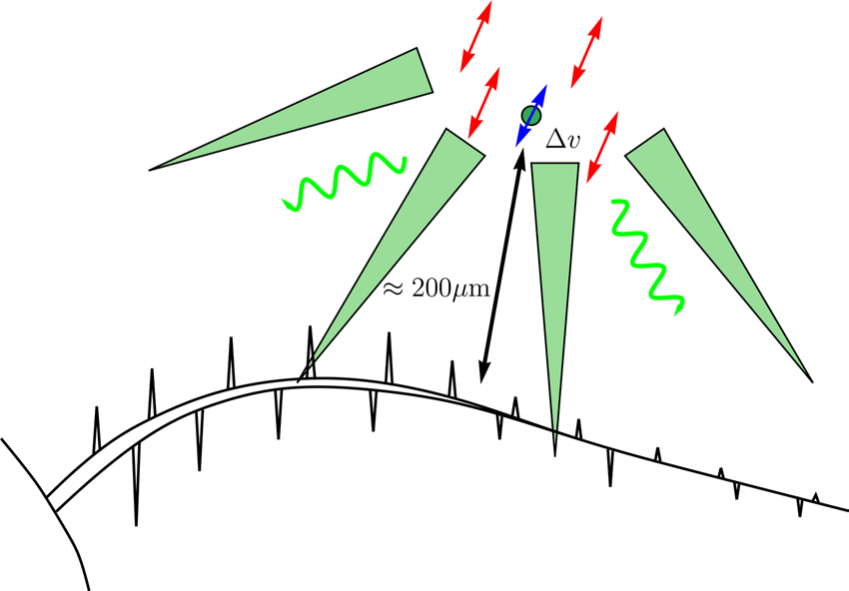
Model of complementary sensing mechanisms of mechanoreception as discussed here and chemoreception following^8,34^. The velocity perturbation Δ*v* propagates in all directions and is damped. The oscillating near-field flow is very efficient in distributing organic compounds from the phycosphere surrounding an algal particle (indicated by the curvy arrows).

Returning to the feeding copepod, not all particles suspended in water are created equal. There are recent debates on the viability of remote chemoreception of algae by copepods that generate feeding currents^46–48^. Here, we propose a new mechanism by which copepods perceive algae in their oscillatory near-field feeding currents. Molecules emitted by organic matter at even very low rates carry information about the quality of the particle as potential foodstuff^49^, see Figure 3 in^8^ for a numerical study and Fig. 4 for a schematic depiction. In laminar flow at low Reynolds number and farther distances it is not obvious how such information reaches the sensory apparatus on the appendages of the animal. Active sensing of volatile organic compounds is well documented in insects^50^, for example by fanning of the wings in moths. In his groundbreaking paper, Aref^51^ indicated mechanisms by which simple periodic motions of rotating stirrers can create chaotic flow patterns, see also^52^. This has now been termed “chaotic advection”. Depending on the precise model for agitator motion, there are frequencies that result in larger areas or volumes of the chaotic advection region. The theoretical considerations of Aref^51^ indicate that too high a beating frequency leads to inefficient stirring, apart from the energetic cost to the animal.

## Methods

### Experimental setup and particle tracking

Adult copepods of the species *Leptodiaptomus sicilis* (S. A. Forbes, 1882) were caught in Lake Michigan at the Fox Point time series station (43°12′N, 87°45′W) near Milwaukee. The animals were stored at 4 °C and used within a day. Copepods were transferred to an observation aquarium with a volume of 125 mL with water at 20 °C and left to acclimatize for 2 h. The untethered copepods were filmed using a Photron camera at 500 Hz together with tracer particles of 15 *μ*m diameter. The concentration of tracer particles was estimated from a frame of the film to be 6 mm^−3^ which is comparable to an environmental algae concentration. The optical system of beam splitters and prisms used for the multi-view films is described in^53^. The digital videos were analysed with CellTrack^54^ and ImageJ^55^ and the data (*x*, *y*, *t*) were exported to Microsoft Excel for further evaluation.

### Data analysis

The numerical procedures have been implemented in the open source package Scilab^56^ and together with the data are available from the authors upon request. The maxilliped was traced at its first joint while we also corrected for the small net motion of the pivot point. To analyse the appendage oscillations we consider the angle that is formed with the bisector of the sweep angle. Tracer particles immersed in the water were traced simultaneously. Cubic polynomials were fitted to the trajectories without self-intersections (18 trajectories out of 35). Cubic polynomials were chosen since all particle trajectories were oriented nearly horizontally and showed relatively little curvature. The density of particles is quite high in order to better visualize the flow field around the copepod. In nature, the encounters are much rarer. To analyse the particle oscillations we used the Discrete Fourier Transform (DFT). The trajectories were smoothened by eliminating all contributions in the Fourier transformed signal that were smaller than 25 % of the peak energy.

## Supplementary information

Supplementary Information

Video 1

Video 2

Video 3

Dataset 1

## Data Availability

The original movies as well as the extracted particle positions are part of this publication as Supplementary Material S3.
