# Supplementary material for: Oscillations in the near-field feeding current of a calanoid copepod are useful for particle sensing
Source: Sci Rep. 2019 Nov 28;9:17742. doi: 10.1038/s41598-019-54264-1 (PMC6882890; doi:10.1038/s41598-019-54264-1)
Supplement: Supplementary file 1 — Supplementary Information [file 41598_2019_54264_MOESM1_ESM.pdf]

Oscillations in the near-field feeding current of a calanoid copepod are useful for particle sensing - Supplementary Material

Carl Giuffre[1], Peter Hinow[2,*], Houshuo Jiang[3], J. Rudi Strickler[4,5]

[1]Department of Mathematics and Computer Science, Adelphi University, Garden City, NY 11530, USA
[2]Department of Mathematical Sciences, University of Wisconsin - Milwaukee, Milwaukee, WI 53201, USA
[3]Department of Applied Ocean Physics and Engineering, Woods Hole Oceanographic Institution, Woods Hole, MA 02543, USA
[4]Department of Biological Sciences, University of Wisconsin - Milwaukee, Milwaukee, WI 53204, USA
[5]Marine Science Institute, University of Texas, Port Aransas, TX 78373, USA
[*]Corresponding author; hinow@uwm.edu

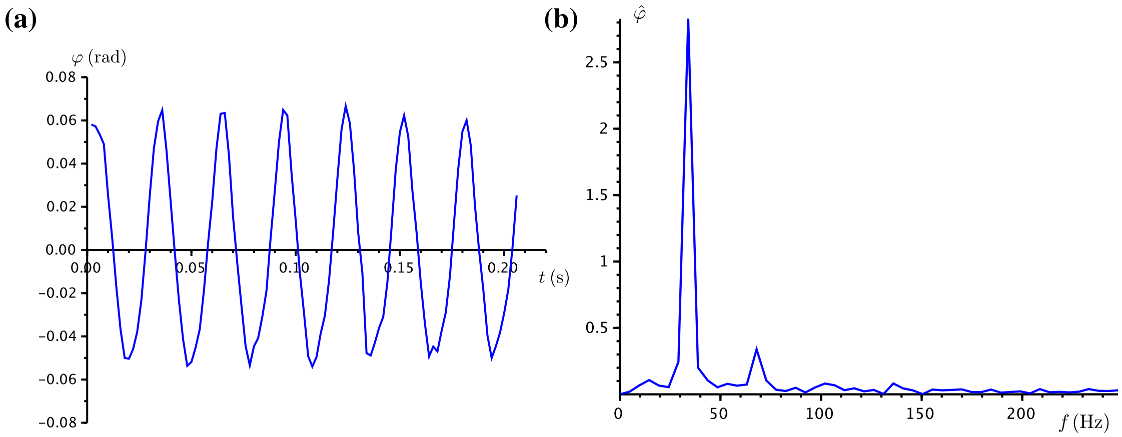

Figure S1. **(a)** The angle that the upper part of the appendage encompasses with the bisector. **(b)** The absolute Fourier spectrum of the angle. Note the two peaks at 34 and 68 Hz, respectively.

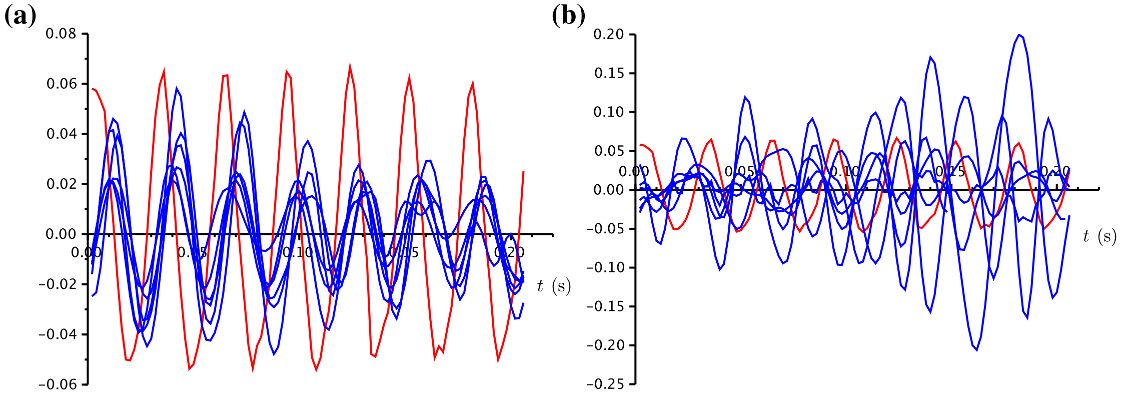

Figure S2. Smoothened particle oscillations from regions A **(a)** and C **(b)**, shown in blue, and the oscillations of the appendage, shown in red. For better visibility the particle oscillations have been amplified by a factor of 20.

S3. The films "Lsicilis29_2_small.mp4" (12.9 MB), "6Lsmall2.mp4" (11.8 MB), "Lsicilis28_short.mp4" (1.3 MB), and the spreadsheet "36_complete_cope_0_103.xls" (388 KB).
